# Are the Mineral Acids Formed in the Mouth?

**Published:** 1871-10

**Authors:** 


### DENTAL REGISTER.

Vol. XXV.] OCTOBER, 1871. [No. 10.

### Communications.

### ARE THE MINERAL ACIDS FORMED IN THE MOUTH?

The author of this article in his reply in the March number of the Register, 1871, charges me with unfairness in the way and manner in which I made use of his paper to serve some purpose entirely different from the original intent.

If such be the case, I was certainly unconscious of such intention. Neither did I intend my criticisms to be carping in any respect, or discreditable to the writer. Neither were they aimed at the chemical formulas—only brought in incidentally. Now, according to Fowne's edition of Chemistry, of 1870, his formulas were mainly correct, there being only two incorrect, and they may have been misprints. My criticisms were in accordance with Youman's edition of 1870, and Fowne's edition of 1867, and all other text books published five or six years since. Fowne's edition of 1867 and Youman's edition of 1870, have been my principle text books for the last three years.

His formula $CaO_3\ C + {}_2HNO = Ca_2\ NO_3 + CO_2 + {}_2HO$ is certainly incorrect, in so far as he only gave one equivalent of Ca first, and ended with two equivalents of Ca. They should both be alike.

His formula $SO_2 + 2\ H_2\ OH_2\ O_4\ S + 2H$ is certainly incorrect. I can not see how he can make use of eight equivalents of H in sulphurous acid, sulphuric acid and water, according to Fowne's edition 1870, or any other work on chemistry.

The writer speaks of the atomic theory as though it was of but recent origin, and had been adopted within the last ten years. The author of this hypothesis, Dr. John Dalton, died in 1844, though he lived to see his new theory universally adopted.

He began this theory in 1803, and published it in the first volume of his *New System of Chemistry*, in 1807, the second volume was published in 1810.

This theory was early investigated and sustained by Drs. Wollaston and Berzelius, who first gave it authority. All the text books since that time are based on that theory. Hare's Chemistry, edition 1840; Kane's edition, 1849; Draper's 1856, contain it, and all others too numerous to mention here.

The author says he has no disposition to enter into a controversy with the writer upon what constitutes a mineral acid, and goes on to give his explanation to the effect that the salts of nitric, hydrochloric, and sulphuric acids, are regarded as minerals, in contradistinction to other salts or compounds. This may be the proper and only distinction. But suppose we take calcium acetate, ferrum acetate, ferrum tartrate, ferrum citrate, calcium oxalate, ferrum prussiate, potassium prussiate, and many others where an organic acid unites with a metalic oxide to form a salt. Are not the above regarded as minerals? They certainly are, as their metallic base determines them to be such; still these facts do not make an inorganic acid a mineral acid by any means. The main difference in the new theory from the old, consists in expressing equivalency in volume instead of weight atomic.

Weight and volume are both equally important. Eight ounces of oxygen and one ounce of hydrogen, by weight, make nine ounces of. water, and always will. Two volumes of hydrogen and one volume of oxygen, make two volumes of watery vapor, or one molecule of water, equal to three volumes. Hence, atomic volume and atomic weight are still understood as before. It will be seen further on, that the elementary equivalency have all been retained without any change, with two exceptions, which are new and old both. The equivalency of water is not given as 3, according to the volumes of gases that form water. Instead, 9 or 18 is still its equivalent—not $H_2 O$ equal 3. According to double atom elemental idea as H = 2 H and O = O, we could have for water 4 atoms of H and 2 of O, making 6 atoms in a molecule of water, but it is not so expressed. The language in chemistry has changed materially, while weights and measures or volumes are the same mainly.

The following pages may not be uninteresting to those who have a taste for chemistry:

*Boston Journal of Chemistry*, May number, 1870. I will give some extracts from the editor's article on the new nomenclature, the article being too long to copy in full.

Says: "In the *Hand Book of Chemistry*, by Messrs. Rolfe & Gillet, we are pleased to find the new nomenclature. It is also found in Roscars *Elementary Treatise*, which we recently noticed."

"It is a singular fact that the main features of this new nomenclature, are not new, but were proposed by Berzelius, half a century ago." "Such terms as ferrous oxide, and ferric oxide, ferrous sulphate, and ferric sulphate, which many imagine to be the invention of the more progressive of living chemists, were first used and advocated by him."

"Nitrous and nitric, sulphurous and sulphuric, have become familiar, as expressing different degrees of oxidation in the case of the acids," and Berzelius wished to apply the same principle of naming the bases and salts.

"Ferrous oxide is more concise than protoxide of iron, and ferric oxide than sesquioxide of iron, and ferrous sulphate is for the same reason better than the protosulphate of iron, and ferricsulphate than sesquisulphate of iron."

"Moreover, as the names ferrous and ferric are derived from the Latin, they will have essentially the same forms in all modern languages, and an approach is thus made to a universal nomenclature."

"Under this new Berzelian method, the names of all the binary compounds are formed in the same way, from their constituents; the name of the electro-positive constituent with the ending, *ic*, being placed before that of the electro negative constituent, which is made to end in *ide*."

"Thus potassium and sulphur, form potassic sulphide, formerly sulphide of potassium, and at an earlier day, sulphuret of potassium; sodium and oxygen form sodic oxide, formerly soda, or oxide of sodium; silver and chlorine form argentic chloride, chloride of silver; lead and iodine, plumbic iodide, iodide of lead, and so on."

"When the same elements form two compounds, the one containing the smaller proportion of the negative element, is distinguised by changing the ending of the name of its positive constituent to *ous*, the ending *ic* being used for the compound containing the larger proportion of the negative element. This is illustrated by the names ferrous oxide, and ferric oxide, of which we have spoken above."

"In the same way we have mercurious and mercuric oxides, cuprous and cupric oxides, of copper; stannous and stannic chlorides, chlorides of tin, etc."

"Calomel is mercurious chloride, and corrosive sublimate is mercuric chloride, and so on."

"When a binary compound contains oxygen, and becomes an acid when united with water, or a salt when united with a base, it is termed an anhydride. Thus one atom of oxygen and two of sulphur, form sulphurous anhydride, formerly sulphurous acid; one of oxygen and three of sulphur, sulphuric anhydride, sulphuric acid."

"The old names are given to the true acids, or the compounds of these anhydrides with water"

"We no longer need use the absurd name anhydrous sulphuric acid, to describe a substance which has no acid properties whatever, and when we speak of sulphuric acid, there can be no doubt which of the two compounds is meant."

"The hydrogen acids or hydracids, are named in the same way as other binary compounds. Thus muriatic acid is hydric chloride, hydrosulphuric or sulphuretted hydrogen, or sulphide of hydrogen, becomes hydric sulphide." "We should not generally speak of water as hydric oxide, any more than we used to call it protoxide of hydrogen."

"The reader will have no difficulty in applying the rule for naming binary compounds to the interpretation of the names of ternary compounds, such as argentic nitrate, sodic phosphate, calcic carbonate, carbonate of lime, plumbic carbonate."

"We will only add that this nomenclature has been adopted by the Chemical Society of London, and is coming to be generally used by the best modern authorities. Some recent writers have adopted thus far only a portion of the system; but consistency and the force of the current will doubtless soon compel them to adopt it in full. A man who persists in speaking of lead nitrate, and sodium sulphate, while he does not hesitate to say ferrous sulphate, or ferric sulphate, is pretty certain eventually to exchange his mixed nomenclature, for a uniform one."

The above, by the editor of the *Journal*, must be admitted by all who are familiar with chemical science, to be a clear and lucid paper on the new nomenclature in relation to the new language proposed. The symbols pertaining to the new order of things, it will be seen farther on, have undergone but little variation, and only in a few instances, which I shall give in full detail.

"In Youman's New Chemistry, edition 1870, may be found many of the latest symbols and formulas as given by Gerhardt and Laurent." Page 274.

| OLD VIEW OF ACIDS. | NEW VIEW OF ACIDS. |
|---|---|
| Phosphoric acid, HO, $PO_3$ | H, $PO_6$, analagous to H, Cy |
| Sulphuric acid, HO, $SO_3$ | H, $SO_4$, " " H, Cl |
| Nitric acid, HO, $NO_5$ | H, $NO_6$, " " H, Br |

Same page—"Hence we arrive at the following definition: An acid is the hydrogen compound of a simple or compound radical which possesses the power of neutralizing bases, its general formula being HR, hydrogen and radicle."

Same page—Later view of Salts—"From this point of view the composition of salts is also simplified; one type of acids gives us also one type of salts. By replacing the hydrogen of chlorohydric acid, HCl, by sodium, we get common salt, NaCl."

By replacing the hydrogen of cyanohydric acid by potassium, we get the salt cyanide of potassium. $KNC_2$.

And so by replacing the hydrogen of sulphionide of hydrogen by iron, we get sulphionide of iron, $FeSO_4$, instead of the old sulphate $FeOSO_3$.

"On this view we may define a salt to be the compound found by replacing the hydrogen of an acid by a metal, and the general formula for a salt is MR., *metal and radicle.*"

Later views of Gerhardt and Laurient. Page 71.

"It is found that the bulk or volume of a grain of hydrogen is the same as that of 14 grains of nitrogen, 35.5 grains of chlorine and 8 grains of bromine. Now, these numbers are precisely the atomic weights of these bodies, so that the same numbers express both atomic weights and specific gravity. But the same bulk of oxygen weighs 16 grains, which is just twice its atomic number, and when the vapor volumes of carbon and sulphur are determined, it is found that to fill the same space takes 12 grains of carbon and 32

of sulphur, these again being just twic e their atomic weights To obtain uniformity, therefore, as well as for other reasons which can not be here stated, the atomic numbers of oxygen, carbon and sulphur, are doubled.

In this way the same numbers are made to express three facts, viz.: atomic weights, specific gravity, and combining volume.

On this view the symbols represent equal volumes of their elements. Hence, the formula for hydrochloric acid, HCl, implies a combination of one volume of hydrogen with one of chlorine. 

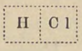

Water is a combination of two volumes of hydrogen with one of oxygen, thus: 

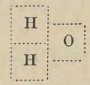

and is written $H_2O$, while ammonia $H_3N$, implies a union of three volumes of hydrogen with one of nitrogen, 

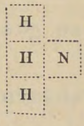

It will be noticed in the case of water, that the doubling of oxygen, is the consequence of the halving of the hydrogen. If we take equal volumes, their weights are as 16 to 1—but, as there are two volumes of hydrogen, the composition becomes $H_2O$, the oxygen being 16."

"There are other reasons for considering the composition of water as more complex than has been formerly supposed, so that without adopting the views of Gerhardt, we may still regard water as $H_2O_2$ instead of HO. Page 196.

Water. Protoxide of Hydrogen.
Sym. HO. Equiv. 9, Sp. G. 1,000.
Chlorohydric Acid. Hydrochloric Acid.
Muriatic Acid. HCl. Page 247.
Nitric Acid. $NO_5$. Page 209.
$KO, NO_5 + 2(HO, SO_3) = (KO, HO, 2SO_3) + HO, NO_5$.

Sulphuric Acid. Sulphuric Anhydride. Page 240.
($SO_3$.) $2SO_3 + HO$. This last Nordhausen.
$SO_3$, HO, or common sulphuric acid. P. 259.
Hydro sulphuric Acid or Sulphuretted Hydrogen, Sulphide of Hydrogen. Page 261.
$FeS + SO_3, HO = FeO, SO_3 + HS$.
Hydrochloric. Page 249.
$NaCl + 2 HO, SO_3 = NaO, HO_2, 2SO_3 + HCl$.
Phosphoric Anhydride. Page 265.
$PO_5$.
Carbonic Anhydride, $CO_2$. Page 218.
$CaO, CO_2 + HCl = CaCl, HO + CO_2$. Page 219.

Again says: Hydrogen gas is not simply H, but $\left.\begin{matrix}H\\H\end{matrix}\right\}$ or $2H_2$, or hydride of hydrogen. Page 191.

Chlorine gas is not Cl, but $\left.\begin{matrix}Cl\\Cl\end{matrix}\right\}$ or chloride of chlorine; while cyanogen is not $C_2N$, but $\left.\begin{matrix}C_2N\\C_2N\end{matrix}\right\}$ or cyanide of cyanogen.

According to this view, the term atom applies to the smallest part of an element which can enter into combination, but which is not known in a separate state. For instance, N is the atom for nitrogen, $\left.\begin{matrix}N\\N\end{matrix}\right\}$ its molecule.

Fownes' Chemistry edition, 1867, page 233.

Binary Theory of Salts—Says: "The great resembling properties between the two classes of saline compounds, the halved and oxy salts, has very naturally led to the supposition that both might possibly be alike constituted, and that the latter instead of being considered compounds of oxides and an acid, might with great propriety, be considered to contain a metal in union with a compound salt radical, having the chemical relations of chlorine and iodine. On this supposition sulphate and nitrate of potassa will be considered in the same manner as chloride of potassium, the compound radical replacing the simple one."

| OLD VIEW. | NEW VIEW. |
|---|---|
| $KO + SO_3$. | $K + SO_4$. |
| $KO + NO_5$. | $K + NO_6$. |

Hydrated sulphuric acid will be like hydrochloric acid a hydrate of a salt radical.

These are some of the new views. Page 239.

Sulphate of potassa, $KO, SO_3$.

Bisulphate of potassa, $KO, SO_3 + HO, SO_3$.

Bisulphate anhydrous, $KO, ClO_5$.

Susquisulphate potassa, $(KO, SO_3) + HO, SO_3$.

Nitrate of potassa, $KO, NO_5$. Page 238.

Bicarbonate potassa, $KO, CO_2 + HO, CO_2$.

TABLE OF EQUIVALENTS. Page 194.

| | |
|---|---|
| Oxygen, - - - - - - - | 8 |
| Hydrogen, - - - - - | 1 |
| Nitrogen, - - - . - - | 14 |
| Carbon, - - - - - | 6 |
| Sulphur, - - - - - - | 8 |
| Phosphorous, - - - - | $10\frac{1}{3}$ |
| Chlorine, - - - - - - | 35.5 |
| Iodine, - - - - - - | $25\frac{2}{5}$ |
| Potassium, - - - - - | 39 |
| Iron, - - - - - - | 28 |
| Copper, - - - - - - | 31.7 |
| Lead, - - - - - - | 103.7 |
| Silver, - - - - - - | 108 |

Fownes' edition, 1867, page 233.

Hydrated sulphuric acid will be like hydrochloric acid, a hydrate of salt radical, $H + SO_4$.

Sulphate of Soda. Page 248.

$NaO, SO_3 + 10 HO$.

Bisulphate of soda, $NaO, SO_3 + HO, S^{\frown}_3 + HO$.

Hyposulphate of soda, $NaO, S_2 O_2$.

Nitrate of soda, $NaO, NO_5$.

Phosphate of soda, 2 NaO, HO, $PO_5 + 24$ HO. Page 219

Tribasic Phosphate, or Sub-phosphate.

3 NaO, $PO_5 + 24$ HO.

Tribasic Phosphate of Soda. Ammonia and water. Microcosmic salt, NaO, $NH_4$ O, HO, $PO_5 + 8$ HO.

Fownes' edition, 1867, page 232.

Bibasic phosphoric acid, 2 NaO, HO + $PO_5$ + 24 HO.

Phosphate of soda, 2 equivalents of base, water, NaO, HO, $PO_5 + 24$ HO. Page 201.

Bin oxide of hydrogen H + $_2$ O, or H $O_2$ or H O2.

Sulphuric acid, S + 3 O or $SO^3$ or $SO_3$.

Hypo sulphuric acid, 2 S + 5O or $S^2 O^5$ or $S_2 O_5$.

Sulphate soda, Na + $SO_4$ or NaO, $SO_3$.

Nitrate of potash, KO + $NO_5$ or KO, $NO_5$.

I might quote more from the same page, but I think I have quoted sufficient to show that here all the old notations or nomenclature are retained, without exception, all within the last five years.

Fownes' edition, 1867, page 140.

5KO, $NO_5 + 2$HO, $SO_3$.

Nitrate of potassa. Sulphuric acid.

KO, $SO_3$, HO, $SO_3$ + HO, $NO_5$.

Bisulphate of potassa. Nitric acid.

Water 4 H O

4 Nitric acid, HO, $NO_5$ Page 143.

Sulphuric acid, $NaO_4$ 2 $SO_2$ + 24 HO = $NO_2$ + 2 HO, S $O_3$.

Hyponitric Acid. Sulphurous water Acid. Binoxide Nitrogen. Hydrated Sulp. acid.

Hydrochloric acid.

NaCl + HO, $SO_3$ = HCl + NaO, $SO_3$. Page 159.

Sulphureted hydrogen.

FeS + HO, $SO_3$ = HS + FeO, $SO_3$. Page 182.

Fowne's Chemistry, edition 1870, page 146.

Says: "The specific gravity of steam or vapor of water, is found by experiment to be 0.625 compared with air, at the same temperature and pressure, or 9 as compared with hydrogen. Now, it has already been shown that water is composed of two volumes of hydrogen and one volume of oxygen, and if the weight of the one volume of hydrogen be taken as unity, that of two volumes of hydrogen (= 2) and one volume of oxygen (= 16) will together make 18, which is the weight of two volumes of watery vapor. Consequently water in the state of vapor consists of two volumes hydrogen and one volume of oxygen, condensed into two volumes."

Page 153. "In symbols, the composition of water and hydrogen dioxide, is thus expressed, Water $OH_2$
Hydrogen dioxide, $O_2\ H_2$"

Page 157. There are five distinct compounds of nitrogen, thus marked and constituted :

| | Composition. | | | |
|---|---|---|---|---|
| | By Weight. | | By Volume. | |
| | Nitrogen. | Oxygen. | Nitrogen. | Oxygen. |
| Nitrogen monoxide nitrogen . | 28 . | 16 | 2 . | 1 |
| Nitrogen dioxide nitrogen . . | 28 . | 32 | 2 . | 2 |
| Nitrogen trioxide and nitrous oxide | 28 . | 48 | 2 . | 3 |
| Nitrogen tetroxide and nitrous oxide | 28 . | 64 | 2 . | 4 |
| Nitrogen pentoxide or nitric oxide . | 28 . | 80 | 2 . | 5 |

Nitrogen monoxide, $N_2\ O$. Nitrogen dioxide, $N_2\ O_2$ .
Nitrogen titroxide, $N_2\ O_3$ , titroxide, $N_2\ O_4$ or $N\ O_2$ .
Nitrogen pentoxide, $N_2\ O_5$ .

| | | | |
|---|---|---|---|
| Univalent elements, or | Monads, | as | H |
| Bivalent " | Dyads | " | $O''$ |
| Trivalent " | Triads | " | $B'''$ |
| Quadrivalent " | Tetrads | " | $C'$ |
| Quinquivalent " | Pentads | " | $P'$ |

Elements of an even equivalency, viz, the dyads, the tetrads and huxads, are called *artiads,* or even equivalency, and the uneven equivalency, viz, the monads, triads and pentads, are generally designated *perissads*, or even equivalency.

Water, $OH_2$ . . . . . H—O—H

Carbonic dioxide, $CO_2$ . . O═C═O

Ammonium chloride, $NH_4$ Cl .

```
      Cl
H     |    H
   >N<
H          H
```

Sulphuric oxide, $SO_3$ . . . .

```
O
‖
S═O
‖
O
```

Sulphuric acid, $SO_4$, $H_2$ . .

```
      O
      ‖
H—O—S—O—H
      ‖
      O
```

Nitric acid, $NO_3$ H . . .

```
O
‖
N—O—H
‖
O
```

Zinc nitrate, $N_2$ $O_6$ Zn .

```
O                O
‖                ‖
N—O—Zn—O—N
‖                ‖
O                O
```

I have not in this instance given the diagrams which are similar to the above.

"The formula for nitric acid indicates that two of the three constituent atoms of oxygen are combined with the nitrogen atoms alone, and are attached to that element by both their units of equivalency, and the third oxygen atom is combined both with nitrogen and with hydrogen."

The above formula for sulphuric acid, is the same found in some of the text books a few years back. Graham's edition, 1858, page 756. Only H instead of $H_2$ being the difference.

$SO_4$ H. This of course is the hydrated acid. On page 295 is the formula for the anhydrous sulphuric acid, eq. 40

or 500. $SO_3$ density of vapor 2,762 the only difference in the old and new being H and $H_2$. According to the volume theory adopted in Fownes' last edition, two of the three atoms of oxygen are united with the nitrogen alone, and the other atom with both N and H. The fact is, this is but half an atom of the acid, if the term is allowable, the hydrogen being an hydroxyl, and in a double or complete molecule; the same thing is repeated, and hence a double hydroxylated nitric oxide, or hydrated nitric acid or pentoxide of nitrogen, and one eq. of water. The only difference between the old and new ideas consists in giving 2 eq. of N instead of one, and $H_2$ the new, being the volume unit instead of quantity unit formerly used by the same author and all others.

In zinc nitrate, as given above, $N_2\ O_6\ Z$, the zinc takes the place of the two equivalents of $H_2$, or the zinc uniting with one eq. of oxygen to form an oxide, hence the anhydrous acid is united with an oxide, and no hydrogen retained. The zinc is not a zinc oxyl as given O — Zn — O but O — Zn instead.

Page 238, Fowne's says: "Thus hydroxyl—O—H is not known in the free state, the actually existing compound containing the same proportions of hydrogen and oxygen being $O_2\ H_2$ or H—O—O—H."

According to the new view this would be a dihydroxyl, but not an atom of water."

Page 297. "Acid potassium carbonate, hydrogen potassium carbonate, or Mono potassic carbonate.

$Ca\ O_3\ K\ H = CO_2\ (KHO.)$"

In other language this formula would be a carbonate of hydroxylate of kalium.

Page 232, says: For the same reason the atoms of elementary bodies rarely exist in the free state, but when separated from any compound, tend to combine other atoms of the same or some other element. Perissad elements like hydrogen, chlorine, nitrogen, and separate from their compounds in pairs, their molecule contains two atoms eq. H II. Artiad elements may unite in groups of two, three, or more, thus: the elements of oxygen in its ordinary state, probably contains two atoms, that of ozone, three atoms, thus: oxygen O = O ozone, 

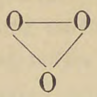

Page 233, says: "That with a few exceptions, the specific gravities, both simple and compound in the gaseous state, are equal to half their molecular weights, or the specific volume the quotient of the molecular weight, by the specific gravities, are equal to 2."

On page 221, the table of equivalents is precisely the same as in the edition of 1867, and other text books, with the exception of two of the elements, namely, phosphorus and iodine. P $10\frac{1}{2}$ or $\frac{31}{3}$. I $25\frac{2}{5}$ or $\frac{127}{5}$.

Page 327. Calcium monoxide lime, CaO. Hydrate of lime, $CaH_2\ O_2$ or CaO, $OH_2$. Calcium dioxide, $CaO_2$. Calcium sulphate, $SO_4$ Ca. Calcium carbonate—marble, $CaO_2$ Ca.

The last edition of the United States Dispensatory, 1870, in the Pharmaceutical Appendix, contains the old notations, and none of the new, for instance, hydrogen protoxide, H O. Acid hydro, sulphurous, or sulphureted hydrogen, H S. Eq. 17.

Tyndal in his Fragments of Science, Page 81, 1871, says, "First, then, you are to remember, that to form water, the proportions by weight of oxygen and hydrogen, are as eight to one, eight ounces of oxygen for example, unite with one of hydrogen, to form nine ounces of water. But if instead of comparing weights, we compare volumes, two volumes of hydrogen unite with one of oxygen to form water. Now, these volumes, and not the weights, express the proportions in which the atoms of hydrogen unite with those of oxygen. In the act of combination, two atoms of hydrogen combine with one of oxygen to form what we call a molecule of water. Every such molecule, is a group of three, two of which are hydrogen and one of oxygen."

Page 88. Says: "The clashing together of one pound of hydrogen and eight pounds of oxygen, to form nine pounds of aqueous vapor, is greater than the clash of a weight of 1,000 tons, falling from a hight of 20 feet against the earth.

The Medical Department of the University of Louisiana, had not adopted the new text books in their lectures last winter, so that it will be seen that the new text books have not been universally adopted as yet.

